# Children as messengers of health knowledge? Impact of health promotion and water infrastructure in schools on facial cleanliness and trachoma in the community

**DOI:** 10.1371/journal.pntd.0009119

**Published:** 2021-02-01

**Authors:** Xinyi Chen, Beatriz Munoz, Harran Mkocha, Meraf A. Wolle, Sheila K. West

**Affiliations:** 1 Dana Center for Preventive Ophthalmology, Johns Hopkins University, Baltimore, Maryland, United States of America; 2 Kongwa Trachoma Project, Kongwa, Tanzania; Universite Laval, CANADA

## Abstract

**Background:**

Health promotion is essential to the SAFE strategy for trachoma elimination. Schools are a valuable venue for health promotion. However, there is little literature about the impact of health education and water infrastructure in schools on facial cleanliness and trachoma in the community. Our study aimed to describe the current state of school health promotion in Kongwa, Tanzania, and to examine the transferability of health messages from schools to the community at large.

**Methodology/Findings:**

A cross-sectional survey was carried out in all 92 villages in Kongwa district, which included 85 primary schools. Data were collected on health messages and water infrastructure in the schools. A random sample of 3084 children aged 0–5 were examined for facial cleanliness in all villages. In 50 villages, a random sample of 50 children aged 1–9 per village were examined for follicular trachoma (TF). Thirty-seven (44.6%) schools had educational materials on face-washing. Fifty (60.2%) schools had a washing station. The presence of a health teacher was correlated with having posters on face washing in classrooms. The presence of face-washing materials was correlated with the availability of washing stations. Neither teachers mentioning face-washing in health curricula nor educational materials in classrooms were associated with clean faces or trachoma in the community. Having a washing station in the school was associated with lower community rates of trachoma.

**Conclusions:**

Primary school health messages and materials on trachoma were not associated with clean faces or lower rates of trachoma in the community. The target audience for primary school health promotion is likely the students themselves, without immediate rippling effects in the community. A long-term perspective should be considered during the implementation of health promotion in schools. The goal of school health promotion should be training the next generation of parents and community health leaders in combatting trachoma.

## Author summary

Trachoma is caused by repeated infection that is spread from person to person via contaminated ocular and nasal discharge. Education on healthy habits such as face washing is essential to the elimination of trachoma. Schools are one place for health promotion programs because so many children can be reached. In previous studies, students showed increased knowledge about trachoma and healthy behavior after engaging in health education programs. We examined if there was evidence that school-based messages were disseminated to the community at large via the primary school students. We found that school health messages did not impact clean faces in pre-school children or trachoma prevalence in the community in general. Having a washing station in the school was associated with lower community rates of trachoma. The data suggested that schoolchildren were not effective messengers of health knowledge and behavior. Primary school health promotion mainly targets students themselves and may not have rippling effects in the community. We should view school health curricula with a long-term perspective as they train the next generation of caregivers and heads of households to raise their own children with healthier habits.

## Introduction

Trachoma is a leading infectious cause of blindness globally, with 137 million people living in trachoma-endemic areas [1]. Trachoma primarily affects the most deprived and vulnerable populations worldwide, particularly in sub-Saharan Africa, the Middle East, and Asia [1]. Trachoma is the result of repeated infection with *Chlamydia trachomatis* (*C*. *trachomatis*), which causes conjunctivitis in young children [2]. *C*. *trachomatis* is transmitted through infected ocular and nasal discharge; it is most prevalent among young children who spread the infection easily to siblings and playmates through direct contact and through fomites [3]. Active trachoma can manifest as follicular trachomatous inflammation (TF) of the upper tarsal conjunctiva. Repeat episodes of infection lead to chronic inflammation, which causes scarring of the conjunctiva (trachomatous scarring). Conjunctival scarring can lead to the in-turning of the eyelid (entropion) as well as the eyelashes (trachomatous trichiasis). Trichiasis can eventually lead to vision impairment and blindness, if left untreated [3].

In order to eliminate trachoma as a public health problem, the World Health Organization recommends a multi-pronged approach known as the SAFE strategy: surgery for trichiasis, antibiotic treatment, facial cleanliness, and environmental improvements [1]. Health promotion is essential to this elimination strategy, especially since it addresses the ‘F’ and ‘E’ components of SAFE [4–8]. Previous health education programs have included radio messages, health extension worker programs, and programs for social organizations; they mainly targeted caregivers and village women with variable penetration rates [9–15]. Health education interventions of modest efforts are deemed insufficient for behavioral changes, while more substantial health education programs were carried out around the time of mass antibiotic administration and the more effective ones are labor-intensive and possibly unsustainable [5, 12, 13]. Longer timeframes are required to change underlying factors that influence behavior, for example social disapproval of families who try to better themselves [5].

In recent years, schools have increasingly been recognized as a valuable venue for trachoma health promotion [8, 16]. Children aged 1–9 are most susceptible to trachoma, and school attendance begins around age 7 [3]. Schools have many mechanisms already built in for sustainable health education including teachers, various educational tools, and positive peer pressure among students. Universal primary education is one of the United Nations Millennium Development Goals, and school enrollment rate has been increasing in most parts of the world [17]. In Tanzania, for example, the gross enrollment rate for primary school has reached 94% in 2018 [18]. Therefore, health messages communicated in a school environment have the potential to reach school-aged children in most households from diverse socio-economic backgrounds. Several studies have found that primary school students showed improved trachoma-related knowledge and face-washing behavior after the implementation of health teaching in schools [19–21]. In one study, the prevalence of clean faces increased among students after a school-based trachoma curriculum [19]. Another study in Ethiopia reported a reduction in the prevalence of moderate and severe trachoma among primary school students following a one-year health education program [22].

It is conceivable that school-aged children can not only develop their own personal hygiene habits through school curricula but also spread hygiene knowledge and practice to the community. Students have been presumed to be able to educate their family, encourage behavioral changes in their families, and help their siblings maintain clean faces, thereby helping to control trachoma in the community [21, 23, 24]. While it sounds like an appealing concept, there is no study to date that investigates the association between school health promotion and clean faces in preschool children and/or lower rates of clinical trachoma in the community.

The first objective of the current study is to characterize health messages and water-related infrastructure in schools in Kongwa district, United Republic of Tanzania. The second objective of this study is to examine the association of trachoma messages and water infrastructure in the schools with facial cleanliness in children under school age in the community, and with trachoma rates in a subset of villages.

## Methods

### Ethics statement

The study was conducted after approval from the Institutional Review Board of the Johns Hopkins University School of Medicine and the Tanzania National Institute for Medical Research. Verbal consent was obtained from the school officials and the guardians of all children in the study. The study adhered to the tenants of the Declaration of Helsinki.

### Setting

Kongwa district, which lies approximately 83 km east of Dodoma (capital city of Tanzania), has a population of approximately 360,000. The average household has 1–2 children aged 5 or under. In 1986, the prevalence of trachoma in pre-school children in Kongwa was reported above 60% [25]. In 2004, the Ministry of Education with the Tanzania Institute of Education created a primary school curriculum on trachoma which was rolled out across the districts. There has been mass drug administration (MDA) in Kongwa district with azithromycin, initially starting in 6 villages in 1999 and growing over time to cover the entire district. The most recent MDA in the district prior to our study was in April, 2016 [26]. The study took place during the winter season from May to September in 2017, in which respiratory illnesses are common in children.

### School survey

A cross-sectional survey of school characteristics was carried out in all villages in Kongwa. Out of the 92 villages, 85 villages had at least one primary school. Children in the other 7 villages went to schools in a nearby village. The interviewer went to at least one primary school in each of the 85 villages. If a village had more than one school, the interviewer went to two randomly selected schools in the village. The interviewer determined if there was a health teacher and a regular teacher available to talk to in every school. If there was a health teacher available, the health teacher was asked if they would be willing to talk about the school health curriculum. The health teacher was not interrupted or prompted by the interviewer. The interviewer noted if the health teacher mentioned hygiene in general (for example, washing and bathing) and if the health teacher mentioned face-washing or clean faces for trachoma. If the school had a regular teacher available, a similar interview was conducted. The interview was supplemented by observations of the classrooms. If none of the classrooms in a school were observable, the interviewer was finished with the school after completion of the teacher questionnaires. For all the observable classrooms, the interviewer noted the presence of any face-washing or clean face materials, and the types of the materials were recorded. The interviewer also observed if there was a washing station in the school, and whether there was water at the station. If no obvious water station was seen, the teacher was asked to show the interviewer the place.

### Facial cleanliness

Facial cleanliness was assessed in children aged 0–5 in randomly selected households in each village. In each mtaa (geographic neighborhood) of a village, a random selection of 4 houses were made. If the village had fewer than 5 mtaas, the number of houses visited per mtaa was increased so that at least 20 households per village were examined. In each household, all children no more than 5 years old in the house or yard during the time of the visit were examined. The interviewer observed the presence of ocular and nasal discharge on children’s faces. Ocular discharge was defined as any clear fluid, cloudy fluid, or dry matter on the lid margin or on the lid. Nasal discharge was defined as wet or dry discharge that was visible outside the nostril when the child was examined in a frontal view without being stared up the nostril. The face of a child was considered unclean if either ocular discharge or nasal discharge was present. The examiner also noted if the child’s face had just been washed by observing if there was water on the face.

### Trachoma prevalence

An impact survey was carried out in Kongwa district in the Fall of 2017, which surveyed 50 randomly selected villages in the district. One grader examined all children aged 1–9 in randomly selected households so that each village had a sample of approximately 50 children. To examine the eye, the grader everted both upper eyelids of each child and scored for the presence of follicular trachoma (TF) using the simplified WHO criteria [27]. TF was recorded as being present if it was seen in either or both eyes.

### Data analyses

Questionnaire data were entered using a custom program in Access. Data were encrypted and sent to Johns Hopkins for analyses with R version 4.0.0 [28]. School characteristics were analyzed using fisher exact tests for contingency tables. If a village had no primary school, the characteristics of the nearby school attended by the children in this village were used in the analysis. One village had no classrooms observed in the first school, so data from the second school in this village were used. The percentage of children aged 0–5 years positive for clean faces was calculated for each village and its association with school characteristics was evaluated with Wilcoxon rank-sum tests or one-way analysis of variance (ANOVA). The associations between school characteristics and village TF prevalence were examined in the subset of 50 villages with Wilcoxon rank-sum tests or ANOVA. Please see S1 Dataset for all data related to this project.

## Results

Among the 85 schools in the study, 68 (80.0%) schools had a health teacher, and 81 (95.3%) schools had a regular teacher available (Table 1). In 2 schools, students were taking exams so no classrooms were observed. Out of the 83 schools with observable classrooms, 37 (44.6%) schools had materials on face-washing or clean faces in classrooms. Among these 37 schools with face-washing materials, 21 (56.8%) schools had only charts or posters in classrooms; 3 (8.1%) schools had small gourds or buckets with a hole and a water spigot used to educate students on hand washing and face washing; 13 (35.1%) schools had both chart/poster and educational gourd/buckets. Fifty (60.2%) of the 83 schools had a washing station, and 47 (94.0%) of the water stations had water.

**Table 1:** Observed characteristics of the 85 schools in Kongwa district*.

Observed Characteristics	N	n	%
School had health teacher	85	68	80.0
Classrooms had materials on face-washing	83**	37	44.6
Classrooms with only chart/poster	37	21	56.8
Classrooms with only educational gourd	37	3	8.1
Classrooms with both educational gourd/bucket and chart/poster	37	13	35.1
School had a washing station	83**	50	60.2
Washing station had water	50	47	94.0

* Only 85 of the 92 villages in Kongwa had their own schools. Children in the other 7 villages attended a nearby school.

** 83 of the 85 schools had observable classrooms; in the other two schools, students were taking tests. If there was no classroom observation, the surveyor was told not to observe if there was any washing station either.

There were no significant differences between the health teachers and regular teachers in unprompted responses about general hygiene or face washing in the school health curricula (Table 2).

**Table 2:** Differences in the interview responses about health curricula between the health teachers (n = 68) and the regular teachers (n = 81).

Responses	Health teacher		Regular teacher		*P-*value
	N	n (%)	N	n (%)	
Mentions general hygiene in curriculum, unprompted	68	68 (100.0)	81	81 (100.0)	1.000
Mentions face-washing for trachoma in curriculum, unprompted	68	59 (86.8)	81	73 (90.1)	0.609

The presence of a health teacher in the school was associated with a chart or poster on face-washing or clean faces in the classrooms (Table 3, *P* = 0.006). The presence of a health teacher was not associated with the presence of a washing station or the availability of water at the washing station. Having face-washing or clean face materials in classrooms was associated with having a washing station in the school (Table 4, *P* = 0.013).

**Table 3:** Comparison of school characteristics by the presence of a health teacher*.

School characteristics	Schools with health teacher	Schools without health teacher	*P-*value
**Face-washing materials in the classroom, n (%)** Yes No	33 (50.0) 33 (50.0)	4 (23.5) 13 (76.4)	0.060
**Chart or poster in the classroom, n (%)** Yes No	32 (48.5) 34 (51.5)	2 (11.8) 15 (88.2)	0.006
**Washing station at the school, n (%)** Yes No	40 (60.6) 26 (39.4)	10 (58.8) 7 (41.2)	1.000
Water at washing station at the school, n (%**) Yes No	37 (92.5) 3 (7.5)	10 (100.0) 0 (0)	1.000

* N = 83 as 83 of the 85 schools had observable classrooms.

** Among those with washing stations.

**Table 4:** Association of face-washing materials in the schools with the presence of a washing station and water at the washing station*.

Characteristics	Face-washing materials present	Face-washing materials absent	*P*-value
**Washing station available, n (%)** Yes No	28 (75.7) 9 (24.3)	22 (47.8) 24 (52.2)	0.013
Water at washing station available, n (%**) Yes No	26 (92.9) 2 (7.1)	21 (95.5) 1 (4.5)	1.000

* 83 out of the 85 schools had classrooms observed; in the other two schools, students were taking tests.

** Among those with washing stations.

A total of 3084 children aged 5 or under from 1798 households were examined for facial cleanliness. Overall, 1546 (50.1%) children had a clean face, and unclean faces were largely the result of having nasal discharge. Only 51 (1.7%) children had just washed their face, so the observations on facial cleanliness were not skewed by recent face washing due to the presence of the interviewer. The percentage of clean faces in children aged 0–5 years in the village was not associated with any of the following: the availability of a health teacher in the school, the mention of face-washing in the curriculum by the health teacher, the mention of face-washing by the regular teacher, the presence of face-washing materials in the classrooms, or the availability of washing stations in the school (Table 5).

**Table 5:** Associations of school characteristics with the percentage of clean faces^#^ in children aged 1–5 years in the village*.

School characteristics	N (villages)	Average percentage of clean faces in the village (SD)	*P-*value
**Health teacher available** Yes No	73 19	50.1 (12.8) 51.7 (14.0)	0.490
**Health teacher mentioned face washing unprompted** Yes No	63 10	50.9 (12.2) 44.9 (15.7)	0.144
**Regular teacher mentioned face washing unprompted** Yes No	79 8	51.3 (12.7) 43.7 (14.7)	0.158
Face-washing materials in classroom^**^ Yes No	38 51	51.2 (12.6) 50.8 (13.0)	0.881
Washing station at school^**^ Yes No	53 36	51.2 (12.6) 50.6 (13.2)	0.802
**Face-washing materials/washing station** Both Only one Neither	29 33 27	51.1 (12.4) 51.5 (13.1) 50.2 (13.3)	0.799

# Calculated as the percentage of observed children in the village who had clean faces.

* Number of observations = 92. For villages without a school, the characteristics of the school that the children attended in a neighboring village were used in analysis.

** In 3 villages, children went to school where classrooms could not be observed due to ongoing exam.

The overall trachoma prevalence in the 50 randomly selected villages was 7.1% but varied by community. Trachoma prevalence in the community was not associated with any of the following: the availability of a health teacher in the schools, the mention of face-washing in the curriculum by the health teacher, the mention of face-washing by the regular teacher, or the presence of face-washing materials in the classrooms (Table 6). However, the availability of a washing station in the school was associated with a lower prevalence of trachoma (*P* = 0.015).

**Table 6:** Associations of school characteristics with prevalence of Trachomatous Follicular Inflammation (TF) in the village*.

School characteristics	N (villages)	Trachoma prevalence (SD)	*P-*value
**Health teacher available** Yes No	42 8	7.5 (6.2) 5.7 (3.9)	0.524
**Health teacher mentioned face washing unprompted** Yes No	38 4	7.8 (6.3) 4.7 (5.9)	0.390
**Regular teacher mentioned face washing unprompted** Yes No	44 4	7.6 (6.0) 3.6 (5.8)	0.204
**Face-washing materials in classroom** Yes No	24 26	7.2 (6.1) 7.3 (5.9)	0.853
**Washing station at school** Yes No	34 16	5.8 (5.3) 10.1 (6.4)	0.015
**Face-washing materials/washing station** Both Only one Neither	19 20 11	6.5 (5.8) 6.2 (5.6) 10.4 (6.3)	0.128

* Number of observations = 50 as data on trachoma prevalence was available in 50 out of the 92 villages. For villages without a school, the characteristics of the school that the children attended in a neighboring village were used in analysis.

## Discussion

In Kongwa district, trachoma prevalence is now low, at 7%. In most villages, the primary schools were found to have some form of health promotion on trachoma. All teachers, both health and regular, described the hygiene component of their curricula unprompted. There was no significant difference between the percentage of health teachers and the percentage of regular teachers who mentioned face-washing for trachoma as being part of the health curriculum unprompted. This indicates teachers’ awareness of and involvement in the school health curriculum as well as good communication between health teachers and regular teachers. It is also crucial for the success of trachoma education to ensure that teachers understand the importance of emphasizing facial cleanliness as part of overall hygiene.

Different teaching methods were employed to reinforce hygiene habits, including chart/posters and gourds/buckets with which demonstrations could be made. International Trachoma Initiative (ITI) recommends active teaching methods to increase the effectiveness of health promotion to school age children [29]. Compared to traditional lectures, active teaching methods augment learning, and make what is being taught more real and more likely for students to remember [29]. Specific active teaching methods recommended by ITI include coming up with short and simple mottos, incorporating health education messages into popular tunes and songs, organizing story-writing competitions with a prize for the best story containing correct health messages, having discussions to reinforce correct health messages, and study tours around village markets to identify health risks and mitigating measures. Many of these active teaching methods have been taken up by health promotion programs in schools [20, 22, 30], although the effectiveness of these approaches in changing long-standing practices in the community and in eliminating trachoma has not been confirmed. In our survey, we did not probe the teachers about how the gourds were used exactly, or if other active teaching methods were employed.

It is interesting that the presence of a health teacher was associated with having a chart/poster in the classroom, and this likely reflects more ready access to hygiene materials on the part of health teachers. Schools that had materials on face-washing in the classroom were also more likely to have a washing station. It is possible that some village leadership devoted more resources to health promotion including investing in educational materials for classrooms and installing water infrastructure in the form of wash stations for the school. Of note, the presence of a health teacher in a school was not associated with the presence of a washing station in that school, suggesting that village leadership likely had more input than health teachers on the decision to make water easily available for hygiene purposes.

Previous studies on school health curricula have focused on the change in trachoma-related knowledge and behavior in schoolchildren themselves [19–22]. Two of these studies have shown significant increases in the percentage of students with clean faces and decreases in the rates of clinical trachoma in students after the implementation of school health programs, although the interpretation of the trachoma observations is complicated by the before-and-after design of both studies, the lack of control groups, and the fact that primary school students with trachoma were identified by health workers and their teachers treated them with antibiotics before the follow-up examination [20, 22].

We focused on a broader impact of the school health program, namely, whether a higher percentage of clean faces in the youngest pre-school-age children were observed in the villages that had more active health programs. We showed that the messages from the school health curriculum are not necessarily translated to clean faces in the pre-school age children. Neither the teachers’ mention of face-washing in the school health curriculum nor the presence of educational materials in a classroom were associated with the percentage of clean faces in the pre-school age group in the community. Null associations were also seen between the school health curricula and trachoma prevalence in the community. This suggests that primary school children may not be successful in disseminating health messages learned in school to their families, helping their younger siblings maintain clean faces, or decreasing the transmission of trachoma.

There are several possible reasons why health messages and behaviors promoted in primary schools did not appear to propagate in the community in general. First of all, primary school students might not have gained understanding equally in all domains of trachoma education, with lack of knowledge in some areas, for example, the relationship between trachoma transmission and some key environmental factors [19]. Moreover, the change in knowledge and behavior in students due to school health curriculum might be insufficient to affect changes at home or impact trachoma transmission [19]. Second, even if students have a comprehensive grasp of trachoma and have clean faces and less trachoma themselves, they are not trained to effectively teach other members of their family and the community. Successful health education in schools entailed training of teachers and intensive discussions on how to troubleshoot and maximize the efficacy of teaching [20]. “Seeing one” and “doing one” do not automatically lead to the ability to “teach one”. Third, it is questionable whether primary school children are culturally permitted to monitor or comment on the behavior of main caregivers in the households and encourage them to maintain healthy habits. Primary school students are not in a position to “train” the elders in their families or to make decisions about the behavior of household members [6]. Furthermore, the disparity between health promotion messages and environmental capacities such as access to water in the households can be limiting in applying the healthy habits [31].

Therefore, health education through school curriculum should be viewed with a long-term perspective. The target audience for primary school health promotion should be mainly students themselves without assuming that there will be immediate rippling effects in the community. However, healthy habits formed in childhood can lay a strong foundation for future health. When these schoolchildren grow older, they may take more responsibility in educating and caring for their younger siblings. Moreover, children of today are the parents and village leaders of the future who will hopefully raise their families with healthier habits.

Another interesting finding of our study is the association between having a washing station at school and lower rates of trachoma in the community. Washing stations in the schools allow students to clean their faces and hands regardless of their home environment. When schoolchildren go back home with clean faces and hands, they are probably less likely to transmit trachoma to their family, although it does not protect them from acquiring *Chlamydia* from other family members. We did examine the rates of trachoma in villages where schools had both washing stations and face washing materials, presuming these schools were the most health conscious, but there was no added benefit above having a face washing stand only. Also, we did not determine when these washing stations were introduced and they might have been present for a long time. If so, the first students who used these washing stations at school could possibly be parents themselves at this point and more likely to have clean faces in their households. It is also plausible that residents in villages whose schools had washing stations had a higher socioeconomic status in general and the association between wealth and lower rates of trachoma has been well demonstrated in prior studies [31, 32]. The reasons behind the association between lower trachoma and a face washing stand at school remain unclear and could represent an ecologic association.

One limitation of the study was the lack of masking of school statuses from the examiners of facial cleanliness. The same set of interviewers for schools conducted the assessment of facial cleanliness in younger children in the households. However, school characteristics were not noted to be correlated with any difference in the prevalence of clean faces, so it is unlikely that there were significant biases. Since the study was carried out in the cold season, the unclean faces might have been driven by upper respiratory illnesses that could cause the smallest children to have persistent nasal discharge despite having faces cleaned. It would have been instructive to conduct a repeat survey in warmer months to see if the prevalence of clean faces changed. Another limitation of the study was the fact that we did not have direct evidence of school-age children living in the studied households, although there were clearly more children than just pre-school-age children in most of the households as the average number of children in each household exceeded the average number of pre-school-age children. In addition, we did not query the teachers about their teaching methods, which might have been informative. We also note that the overall prevalence of trachoma in this district has fallen from over 60% in 1986 to 7% in 2017 [25], so we cannot presume that the school health program did not have any contribution over that time period; we can only suggest that the current variation in trachoma prevalence or clean face prevalence at the village level is not associated with elements of the school curricula at the present time.

In summary, our study found that washing stations at school were associated with lower community rates of trachoma, and further research is warranted to understand the association. In general, primary school health curricula on trachoma were not significantly correlated with the prevalence of clean faces in pre-school children or trachoma in the community at large in Kongwa, Tanzania, where the overall estimate of trachoma was 7%. A long-term perspective should be considered at this point during the implementation of health promotion in schools. The goal of school health curricula should be training the next generation of health leaders in the combat against trachoma. Future studies can more closely examine barriers to family behavioral change despite school health messages.

## Supporting information

S1 Dataset Data collected and used for analysis in the study. (XLSX)
